# Disulphide maintenance systems and proteostasis

**DOI:** 10.1098/rstb.2025.0280

**Published:** 2026-10-01

**Authors:** James D. West

**Affiliations:** Biochemistry & Molecular Biology Program, Departments of Biology and Chemistry, The College of Wooster, Wooster, OH 44691, USA

**Keywords:** disulphide, thioredoxin, protein disulphide isomerase, Mia40, chaperone

## Abstract

Protein disulphide formation between cysteine residues impacts protein folding and function, yet the context in which it occurs often determines whether it is beneficial or detrimental. To ensure proper disulphide status, organisms have evolved compartment-specific disulphide maintenance systems. Some of these, like the thioredoxin and glutaredoxin systems, promote disulphide reduction in oxidized substrates, whereas others, like the protein disulphide isomerase, Mia40 and Dsb systems, primarily oversee proper structural disulphide formation within client proteins. Here, I review how these disulphide maintenance systems operate and explore how they fit within the proteostasis network. Each system functions in helping proteins achieve and/or retain their functional states. Along these lines, thioredoxins can prevent aggregation of some proteins and, working with holdases, regulate recovery from protein misfolding during oxidative stress. Separately, protein disulphide isomerases work collaboratively with core molecular chaperones (e.g. Hsp70 family members) to aid in protein folding, enabling the correct formation of structural disulphides in clients. In addition, some disulphide maintenance enzymes influence protein degradation of improperly folded proteins by the proteasome. This review highlights several key ways that protein disulphide maintenance and proteostasis go hand-in-hand.

This article is part of the Theo Murphy meeting issue ‘ProteostaSys: a systems view of proteostasis’.

## 1. Oxidation of cysteine residues in proteins

Oxidation of cysteine residues has long been considered to play a role in protein structure and function, dating back to early studies of folding of model proteins like RNase A [1]. However, how cysteine oxidation influences protein folding and function has since been found to be context-dependent, especially from a cellular localization standpoint. Some compartments like the cytosol are reducing in nature, whereas other compartments, like the periplasm in Gram-negative bacteria and the endoplasmic reticulum (ER) in eukaryotes, are more oxidizing [2,3]. Likewise, environmental conditions can alter redox homeostasis and influence cysteine oxidation. For instance, imbalanced oxidant production caused by aberrant mitochondrial electron transport, immune responses, receipt of extracellular signals or exposure to radiation or various small molecules can alter redox homeostasis and cause oxidative stress [4]. Such stress can lead to protein oxidation on cysteine residues, often altering protein function [5]. Conversely, perturbing ER redox homeostasis can disrupt proper protein oxidation and trigger localized protein misfolding [6]. Thus, in some cases, excessive oxidation is undesirable and leads to protein misfolding and malfunction; however, in other instances, protein oxidation on cysteine residues stabilizes the protein's native state and enables its function. As a result, regulation of cysteine oxidation is closely tied to human health, and its impairment is thought to contribute to various age-related diseases, protein conformational diseases and microbial virulence [7–10].

Structural microenvironments within a folded protein or a folding intermediate can influence the ionizability, reactivity and oxidizable nature of accessible cysteine residues [11,12]. Stated differently, depending on its location within a protein, a cysteine's thiol can be deprotonated to form a thiolate, thereby increasing its nucleophilicity; this enhanced reactivity can, in some cases, render it susceptible to oxidation (figure 1). With peroxides, a cysteine is oxidized on the thiolate initially to form a sulphenic acid, which has several possible fates (figure 1) [11–13]. The sulphenic acid can react with the peptide backbone to yield a sulphenamide, condense with another thiol group to form a disulphide, react with hydrogen sulphide (H_2_S) to produce a persulphide, or undergo further oxidation (hyperoxidation) to form sulphinic and sulphonic acids (figure 1) [11–13]. With disulphide formation, such bonds either form between the oxidized cysteine and another cysteine in a protein or with a thiol-containing molecule like glutathione [11–13]. Some disulphides are formed during catalysis (e.g. in various oxidant defence and metabolic enzymes), whereas other disulphides play regulatory or structural roles [14,15]. In this review, I focus principally on systems that facilitate disulphide reduction, introduction and rearrangement in proteins and the ways they influence protein quality control.

**Figure 1.**
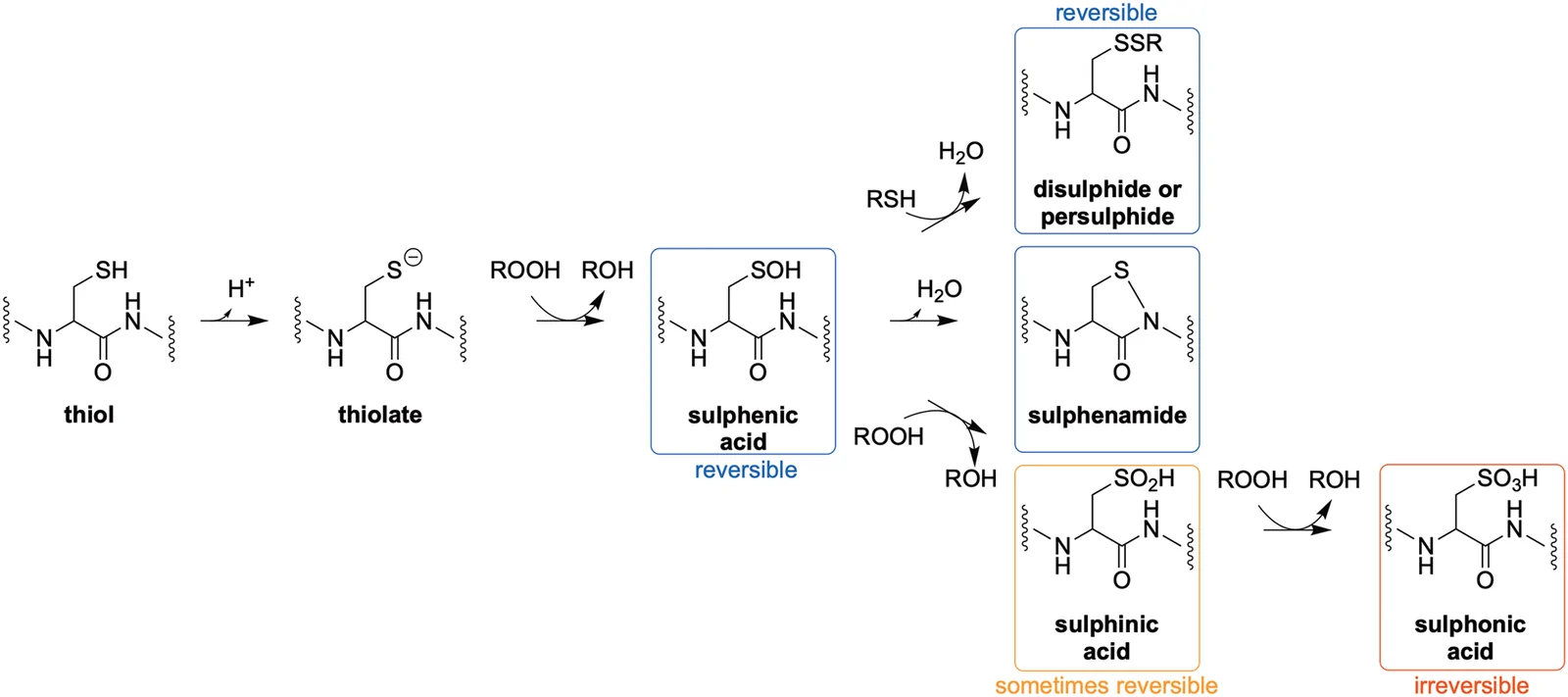
Common oxidized forms of cysteines. Cysteine susceptibility to oxidation may be enhanced by deprotonation of the thiol to form a thiolate, which reacts with peroxides to produce a sulphenic acid intermediate. The sulphenic acid can either undergo condensation with another thiol to produce a disulphide or with the peptide backbone to yield a sulphenamide. Alternatively, the sulphenic acid may be further oxidized to a sulphinic or sulphonic acid. Reversible oxoforms are denoted in a blue box; conditionally reversible and irreversible oxoforms are depicted in an orange or red box, respectively.

## 2. General features of disulphide maintenance systems

Subcellular compartments contain unique disulphide maintenance systems that contribute to the function of their client/substrate proteins. Their function commonly mirrors the redox environment of the compartment, with systems in reducing compartments (e.g. cytosol, nucleus and mitochondrial matrix) functioning in disulphide reduction and systems in oxidizing compartments (e.g. ER lumen and bacterial periplasm) largely functioning in disulphide formation and rearrangement. Disulphide reductases found in reducing compartments include thioredoxins and glutaredoxins [16], whereas protein disulphide isomerases (PDIs), Mia40 (mitochondrial import and assembly protein 40, also known as CHCHD4) and disulphide bond-forming (Dsb) enzymes principally carry out disulphide introduction (and sometimes rearrangement) in the ER, the mitochondrial intermembrane space or the periplasm of Gram-negative bacteria, respectively [3,17,18] (figure 2). These systems operate using thiol-disulphide exchange mechanisms with substrates [19], and all, with the exception of Mia40, are evolutionarily related, belonging to the thioredoxin superfamily [16–18,20,21]. These disulphide maintenance enzymes often partner directly with flavoproteins or proteins containing other redox-active cofactors, allowing them to contribute to protein quality control through stepwise redox relays [3,22].

**Figure 2.**
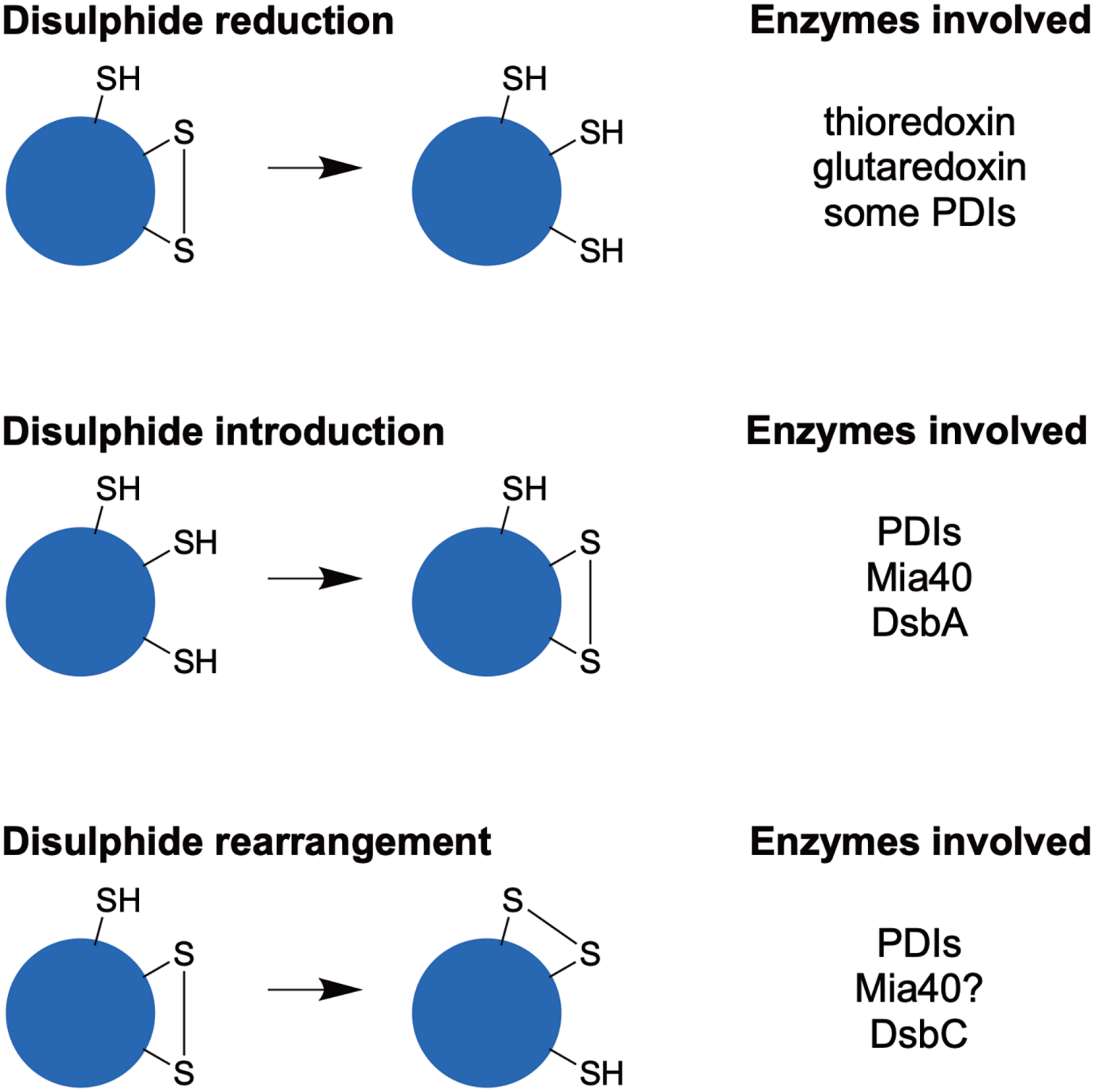
Thiol-disulphide exchange chemistries carried out by key disulphide maintenance enzymes. Some disulphide maintenance enzymes can catalyse disulphide reduction, introduction and/or rearrangement in substrates/client proteins. The enzymes that perform each type of disulphide chemistry are noted on the right.

### (a) Disulphide reduction by thioredoxins and glutaredoxins

Thioredoxins reduce disulphide bonds in substrates by partnering with thioredoxin reductase [23–25]. Initially, a non-native disulphide in a substrate is recognized via weak forces of attraction (e.g. hydrophobic and electrostatic interactions) [26–31]. Entropy appears to be the principal thermodynamic determinant in substrate recognition [31]. Upon substrate binding, the thiol group on the *N*-terminal cysteine in thioredoxin's CXXC active site motif attacks a sulphur in the substrate disulphide to form a mixed disulphide intermediate between the proteins [24,25]. Subsequently, the thiol group of the *C*-terminal cysteine in thioredoxin attacks the sulphur on the *N*-terminal cysteine to displace the reduced substrate, forming an intramolecular disulphide between thioredoxin's active site cysteine pair. Thioredoxin then undergoes a thiol-disulphide exchange with thioredoxin reductase to be returned to its reduced, active form [32]. While two distinct forms of thioredoxin reductase have arisen throughout evolution, both enzymes are dimeric, NADPH-dependent flavoproteins [32].

Glutaredoxins constitute another branch of disulphide reductases within the thioredoxin superfamily. Although less-characterized than their thioredoxin counterparts, many glutaredoxins function similarly to thioredoxins [16]. However, their roles are more highly varied and their mechanisms more complex [33–35]. For instance, class I glutaredoxins can reduce both *S*-glutathionylated proteins and protein–protein disulphides via several glutathione-dependent mechanisms, each of which ultimately produces glutathione disulphide. Although class I glutaredoxins are thought to mainly reduce glutathionylated proteins, functional redundancy between thioredoxins and this subfamily of glutaredoxins has been proposed [36,37]. By contrast, class II family members have an alternative active site structure and, as a result, play a limited role in disulphide reduction and participate instead in iron–sulphur cluster assembly [38].

### (b) Disulphide introduction and rearrangement by protein disulphide isomerases, Mia40 and disulphide bond-forming proteins

The more oxidizing compartments of cells contain designated machineries that introduce structural disulphides into proteins upon their translocation from the cytosol. These enzymes sometimes carry out disulphide rearrangement if the original oxidation does not result in a properly folded protein. Some also act as disulphide reductases in particular instances.

PDIs arguably represent the most diverse and structurally varied class of thioredoxin superfamily members, with baker's yeast expressing five different isoforms and humans expressing 21 isoforms [39,40]. Most PDIs localize to the ER, where they collaborate with the ER oxidase Ero1, a flavoprotein that aids in structural disulphide formation [6,39]. PDIs undergo direct thiol-disulphide exchange with Ero1 (which, while consuming O_2_ to produce an exchangeable disulphide, also generates H_2_O_2_) [6]. PDIs thereby acquire disulphide bonds that can be exchanged into substrate proteins translocated into the ER lumen [6]. Alternatively, reduced PDIs may participate in disulphide rearrangement for improperly folded proteins and/or protein reduction of terminally misfolded proteins [41,42]. Many PDI family members contain two or more canonical CXXC motifs (often CGHC) in their thioredoxin domains, although this motif is mutated to CXXS in some isoforms [40]. Moreover, some family members only possess one redox-active thioredoxin domain [40]. In addition, some family members have transmembrane and/or other functional domains (e.g. DnaJ domains, etc.) that suggest specialized roles in disulphide maintenance and proteostasis [8]. Indeed, only a few PDI family members are considered to be true functional orthologues of one another, whereas other family members in the same organism are thought to have more niche-specific, paralogous roles, imparted by their varied domain structures [43,44].

Similar to PDIs, the enzyme Mia40 introduces structural disulphides into proteins translocated into the mitochondrial intermembrane space [45–47]. Mia40 is directly oxidized on its CXC active site motif by a partner flavoprotein, Erv1 (also known as ALR in humans), that transfers electrons from Mia40 to downstream partners (e.g. cytochrome *c*) [45,47]. Unlike the PDIs and other thioredoxin superfamily members, Mia40 preferentially recognizes specific sequences in substrates for oxidation (e.g. CX9C and CX3C motifs) [48]. This sequence specificity has enabled identification of putative Mia40 clients using bioinformatics [49–51], although some Mia40 substrates lack these sequence motifs [48,52]. While the role of Mia40 in substrate oxidation is established, a role in disulphide rearrangement appears less certain [53,54]. Nonetheless, Mia40 serves a key function in enabling oxidative protein folding for proteins transported into the intermembrane space.

An analogous oxidative folding system to that in the ER or intermembrane space carries out structural disulphide formation in the periplasm of Gram-negative bacteria. The proteins involved in these reactions belong to the DsbA and DsbC branches of the thioredoxin superfamily, with DsbA primarily functioning as an oxidase for its clients [9,55]. DsbC isomerizes disulphides in improperly folded proteins after oxidation by DsbA [9,55]. DsbA partners with the ubiquinone-interacting protein DsbB to become oxidized, whereas DsbC partners with DsbD and the cytosolic thioredoxin system to be maintained in its reduced, isomerase-competent, state [9,56]. Additional Dsb proteins like DsbG play redox roles in the periplasm [57], and similar systems that localize to the periphery of Gram-positive bacteria are currently being characterized [58].

## 3. Active site features and functions of thioredoxin superfamily members

Numerous thioredoxin superfamily members have been identified from organisms across biological domains in recent years, presenting the challenge of determining the activity/activities that these proteins possess. Certain active site motifs have been closely associated with thioredoxin (CGPC), glutaredoxin (CP/SYC), PDI (CGHC), DsbA (CPHC) and DsbC (CGYC) [19]. However, new superfamily members often have variant active site sequences, necessitating screening approaches, mostly in microbial systems, to help guide whether they function in disulphide reduction, transfer and/or rearrangement [59–61]. The results from these studies reveal that active site motif sequences are not always reliable predictors of function. For instance, several periplasm-localized thioredoxin variants bearing mutated catalytic motifs complement phenotypes associated with DsbA deletion, with the consensus CPHC motif from DsbA being the most active [59]. Likewise, a recent metagenomic screen of thioredoxin superfamily members revealed that, in many cases, electrostatic surface maps instead of active site motifs correlate better with biological function [60]. Separately, the yeast thioredoxin Trx2 exhibits a broad tolerance for substitutions at the conserved glycine and lysine in the WCGPCK thioredoxin catalytic motif, implying that variability is allowed in what historically was considered a stringent sequence requirement [61]. A key area moving forward will be to define additional molecular determinants within thioredoxin superfamily members that confer their disulphide maintenance activities.

## 4. Identifying putative redox partners of disulphide maintenance enzymes

Since disulphide maintenance enzymes form transient disulphides with their redox partners, a common challenge encountered has been isolating their substrate/client proteins. Several approaches have been developed to capture these enzymes with interaction partners, allowing for identification of potential substrates (reviewed in [19] and [62]). Kinetic trapping mutants (those that lack a resolving cysteine in the redox motif) are commonly used to capture intermediates formed between disulphide maintenance enzymes and substrates (reviewed in [19,62,63]). These intermediates are often in low abundance and are potentially reversible during sample workup. Moreover, expression of the trapping mutant may have adverse biological effects, especially if the enzyme being mutated is essential. To circumvent these problems, disulphide-linked complexes can be trapped using this method *ex vivo*. Alternatively, several laboratories have used small thiol-reactive cross-linkers, such as divinyl sulphone, to trap disulphide maintenance enzymes complexed with putative redox partners [64–70]. Although this approach probably does not capture all disulphide-based interactions and relies on non-redox chemistry, it is irreversible, can capture interactions *in vivo* and avoids extensive genetic manipulation of the enzyme. These approaches have revealed numerous interaction partners of disulphide maintenance enzymes and, in some cases, have solidified connections to the broader proteostasis network. For instance, both kinetic trapping and cross-linking approaches have been useful in establishing a link among individual PDIs, upstream oxidation machinery, other core chaperones, protein glycosylation enzymes and components of the ER-associated degradation (ERAD) system [67,68,71,72]. Since many disulphide maintenance enzymes remain uncharacterized, these approaches will probably prove useful in uncovering how they contribute to proteostasis.

## 5. Disulphide maintenance enzymes and the proteostasis network

In addition to collaborating with their redox relay partners, enzymes that oversee disulphide bond reduction, formation and rearrangement often interact with several components of the proteostasis network. This network encompasses the machineries responsible for all aspects of a protein's life cycle, from translation to folding into a native state to degradation [73,74]. As noted below, the different disulphide maintenance enzymes act as holdases to prevent protein aggregation, interact with specific ATP-dependent foldases (e.g. compartment-specific Hsp70/DnaK proteins) and aid in misfolded protein clearance, suggesting multiple touchpoints on protein quality control. Early links between disulphide formation and proteostasis were established by studying stress-responsive gene expression, where oxidants that trigger disulphide formation were found to activate the heat-shock transcription factor Hsf1 and upregulate numerous molecular chaperone genes [75,76]. Conversely, exposing cells to disulphide reductants like dithiothreitol initiates an ER stress response, indicating impaired secretory pathway proteostasis [77]. Not surprisingly, the enzymes that facilitate disulphide maintenance in these compartments—thioredoxins and PDIs—arguably have the clearest links with proteostasis at present. Below, I highlight the different ways that disulphide maintenance enzymes contribute to proteostasis.

### (a) Functions of thioredoxins in proteostasis

Thioredoxins probably contribute most directly to proteostasis by reducing substrates that contain non-native disulphide bonds (figure 3). In support of this idea, organisms lacking thioredoxins (or their corresponding reductases) exhibit elevated heat-shock responsive gene expression, suggesting elevated levels of overall misfolding. In *Saccharomyces cerevisiae*, for example, strains lacking the cytosolic thioredoxins Trx1 and Trx2 or their reductase Trr1 have a constitutively active heat-shock response [78]. These findings mirror those in *Escherichia coli*, where combined deletion of thioredoxin reductase (TrxB) and glutathione reductase (Gor) increased activity of the heat-shock response regulator σ32 [79]. It is currently unclear how the heat-shock response is repressed by thioredoxins, although several possibilities exist. Thioredoxins could keep particular foldases like Hsp70 in a reduced (and active) state, thereby allowing them to act as direct repressors of Hsf1 [80]. Alternatively, thioredoxins may safeguard the proteome from general misfolding and aggregation [80]. Indeed, yeast lacking a cytosolic thioredoxin system exhibits an increase in general protein aggregation [78]. In a similar vein, thioredoxins are thought to play a role in ribosomal assembly, as evidenced by enhanced ribosomal protein aggregation in yeast lacking Trx1 and Trx2 during oxidative stress [81].

**Figure 3.**
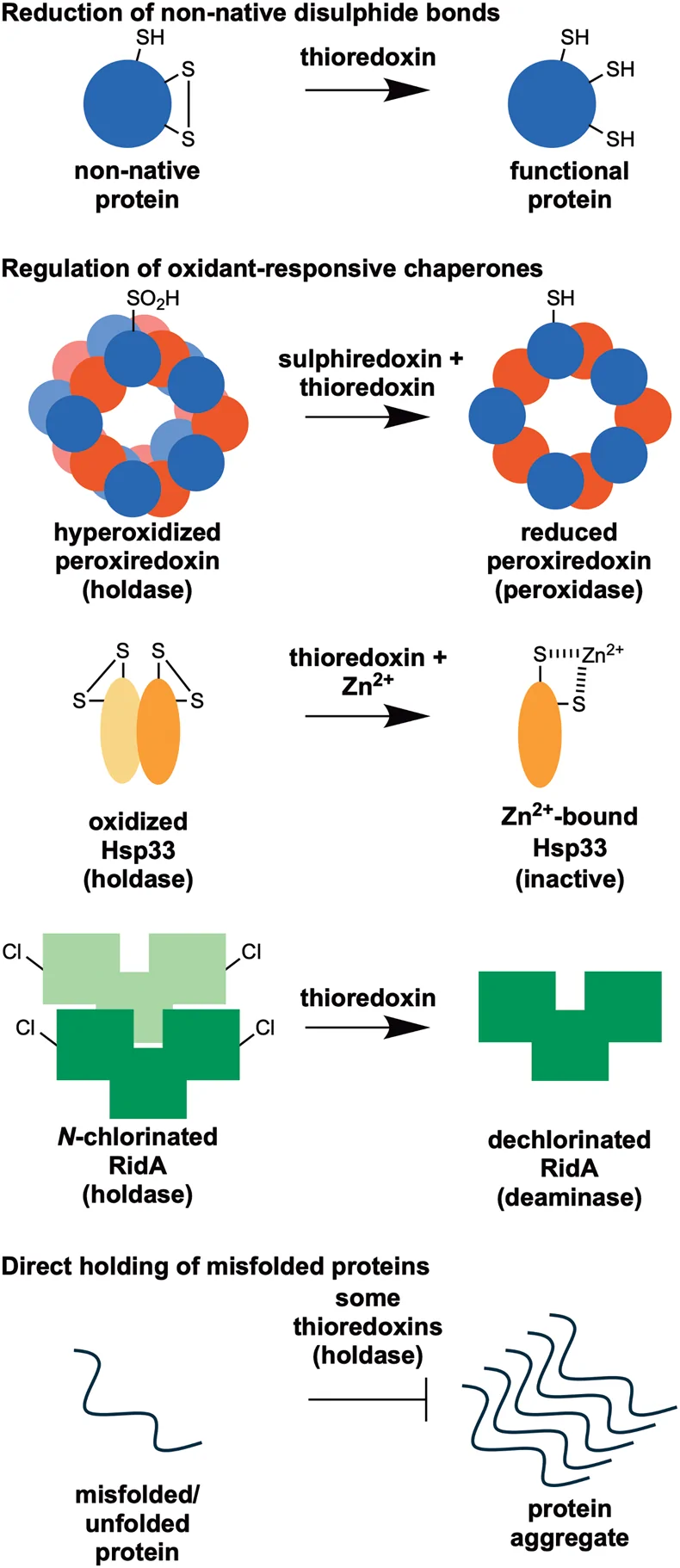
Involvement of canonical thioredoxins in proteostasis. Thioredoxins reduce non-native disulphide bonds in substrates to restore them to their functional state, regulate the activities of oxidant-activated holdases (like hyperoxidized peroxiredoxins, Hsp33 and RidA, with oxidative modifications shown in a simplified form) and, in certain instances, exhibit their own holdase activity to prevent aggregation.

Thioredoxins also contribute to proteostasis by controlling the activity of oxidant-activated chaperones (figure 3). For example, eukaryotic thioredoxins partner with peroxiredoxins and sulphiredoxins to influence the holdase activity of hyperoxidized peroxiredoxins. During oxidative stress, peroxiredoxins can become hyperoxidized to form a sulphinic acid on their peroxidatic cysteine [82,83]. Sulphinic acid formation inactivates the peroxiredoxin's peroxidase activity and, instead, causes this decameric enzyme to oligomerize into a high molecular weight complex with holdase activity [84,85]. Hyperoxidized peroxiredoxins can also form complexes with other chaperones like Hsp70 [86]. Such holdase activity of peroxiredoxins and association with other chaperones probably protects cells against protein aggregation, as ATP levels and, therefore, the activities of ATP-dependent foldases decrease during oxidative stress [5]. Yet, cells have evolved a thioredoxin-dependent regulatory system that turns off peroxiredoxin chaperone activity. This regulation employs a thioredoxin partner, sulphiredoxin, to bring about the switch from holdase to peroxidase [87]. Sulphiredoxin first reduces the hyperoxidized cysteine on the peroxiredoxin to a sulphenic acid in an ATP-dependent manner [88,89]. Thioredoxin can convert both the oxidized sulphiredoxin and peroxiredoxin back to their reduced states [90]. Thus, thioredoxin aids in turning off peroxiredoxin holdase activity during recovery from oxidative stress. Whether thioredoxin regulates other oxidant-activated chaperones in eukaryotes (e.g. Get3) remains to be determined [91], although it does inactivate the chaperone function of Hsp33, an analogous holdase in bacteria, restoring the protein to a Zn^2+^-bound monomer from an oxidized dimer [92]. Likewise, RidA, another bacterial protein that is activated by hypochlorite to form a multimeric holdase complex, is inactivated by thioredoxin via dechlorination [93].

Some thioredoxin family members function as chaperones themselves, exhibiting holdase activity (figure 3) [21,94]. In *Arabidopsis thaliana*, the thioredoxin Trx-h3 exists in two forms—a low molecular weight disulphide reductase and an oligomeric form that possesses redox-independent chaperone activity [95]. Likewise, two tobacco plastid thioredoxins (Trx f and m) act as disulphide reductases but exhibit holdase activity under specific conditions [96]. In *Trypanosoma brucei*, the mitochondrial thioredoxin Trx2 lacks disulphide reductase activity but blocks thermal aggregation of model substrates [97]. The chaperone activity of *Trypanosoma* Trx2 is most pronounced in its reduced state, although this activity is retained when mutating its cysteines to serine [97]. Thioredoxins can sometimes contribute to the folding and function of cysteine-free clients. For instance, the *E. coli* thioredoxin TrxA enables the function of the ADP-ribosyltransferase TreX, a protein that lacks cysteines [98]. Since chaperone activity is not currently considered a universal feature for all thioredoxins, further work is needed to define the structural features within thioredoxins that contribute to this property.

While a role for canonical thioredoxins in protein degradation has not been clearly established, thioredoxin-related proteins that exhibit thioredoxin activity *in vitro* stably associate with the proteasome and contribute to protein turnover. For example, the thioredoxin relative TXNL1/TRP32 exhibits disulphide reductase activity and associates with the 26S proteasome, specifically interacting with the deubiquitinase Rpn11, the ubiquitin-binding protein Rpn10, and the scaffold Rpn2 in the 19S regulatory lid [99–102]. However, the role of redox chemistry in TXNL1 function needs further study to understand whether it plays a role in protein degradation.

Collectively, numerous studies indicate roles for thioredoxin and related proteins in proteostasis, and they underscore the versatility of thioredoxins in safeguarding the proteome in reducing compartments like the cytosol. Similarly, thioredoxin superfamily members that reside in more oxidizing compartments contribute to multiple aspects of proteostasis, as is perhaps best exemplified by PDIs in the ER.

### (b) Functions of protein disulphide isomerases in proteostasis

PDIs aid in structural disulphide formation in proteins entering the secretory pathway. They chaperone proteins in a redox-dependent manner by introducing or rearranging disulphide bonds in substrates to promote folding, unfolding or, sometimes, aggregation [103–105]. Some PDIs also function as redox-independent holdases or acquire such activity by phosphorylation during ER stress (figure 4) [106,107]. As a result, PDIs contribute to protein structural stability, even prolonging the half-lives of terminally misfolded proteins in the ER lumen [108]. Individual PDIs also partner with other chaperones to fold client proteins (figure 4) [109,110]. Some PDIs form large complexes with core chaperones (like the ER-resident Hsp70 BiP) [111], suggesting that specific PDIs are targeted to particular clients through these associations [71]. For instance, in the presence of nascent polypeptides, human PDIA1 complexes with other chaperones and proteostasis factors, including BiP, GRP94, ERdj3, cyclophilin B and UDP-glycosyltransferase [111]. Relatedly, human PDIA6 forms a scaffold that recruits a multichaperone complex consisting of BiP, ERdj3, PDIA1, PDIA3 and GRP94 into phase-separated condensates [112,113]. Once formed, these multichaperone complexes enhance folding of nascent proteins entering the ER lumen [111,112], although it remains to be determined if they influence other aspects of proteostasis, like protein degradation.

**Figure 4.**
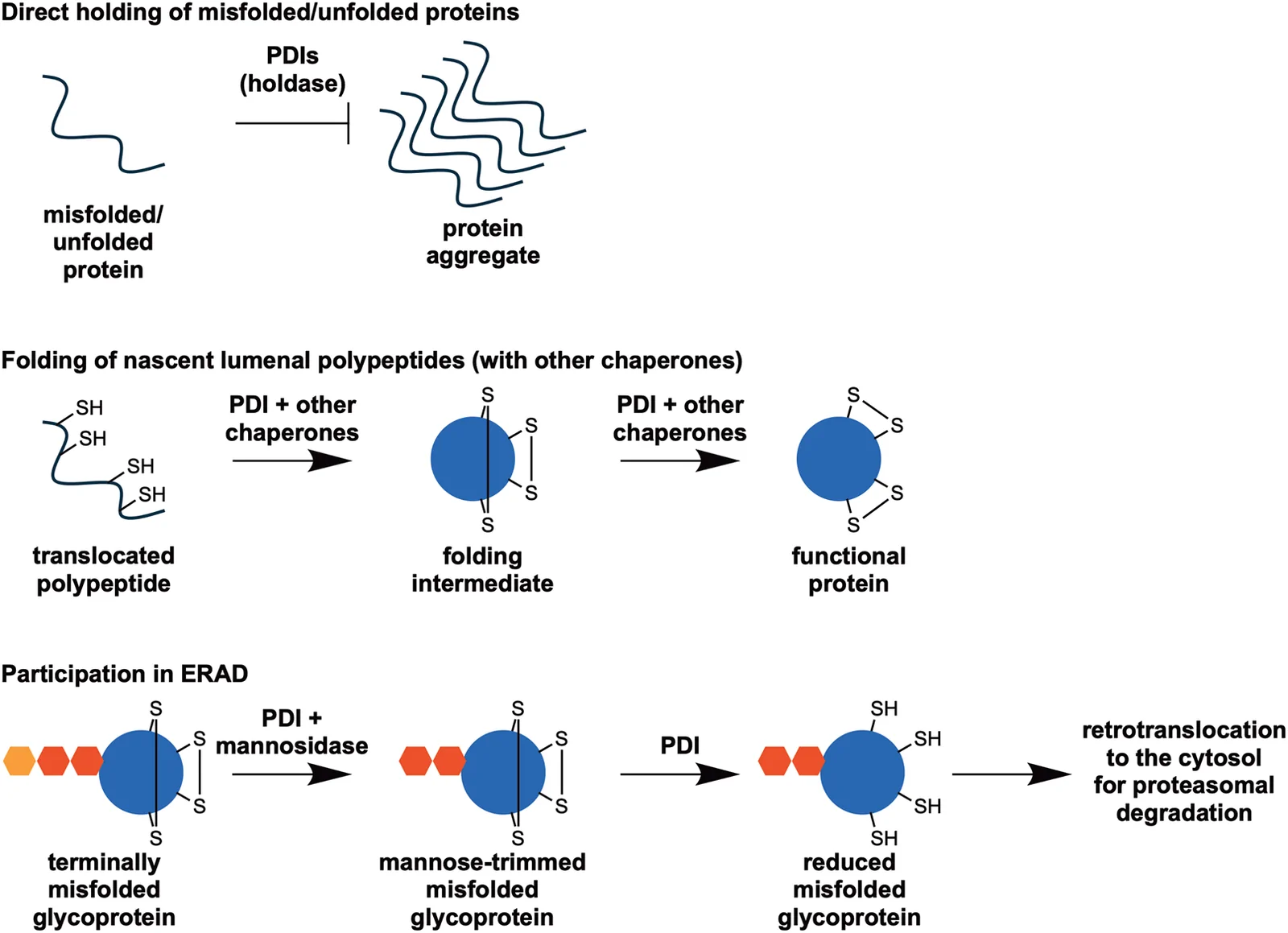
Involvement of PDIs in proteostasis. PDIs can hold thermally denatured proteins to prevent aggregation. For polypeptides imported into the ER lumen, they collaborate with other chaperones to ensure proper structural disulphide formation and folding. They also partner with mannosidases to bring about sugar trimming and reduce disulphides in ERAD substrates before export to the cytosol for degradation.

With regard to protein turnover, several PDI family members partner with the ERAD machinery to clear misfolded proteins. First, many PDIs exhibit holdase activity that can limit protein aggregation [105,114]. While the mechanism for how this contributes to ERAD is unclear, PDI holdase activity impacts the clearance rate of some ERAD substrates [115]. Direct links between specific PDIs and individual ERAD system proteins have also been established. For instance, in baker's yeast, Pdi1 complexes with the mannosidase enzyme Mnl1/Htm1, which hydrolyses a single sugar residue on protein substrates and thereby exposes a key signal for the export of misfolded glycoproteins (figure 4) [116–119]. Following glycan trimming, Pdi1 reduces the client's disulphides before its export into the cytosol [120]. Similarly, the human PDI ERp46 and the mannose-trimming enzyme EDEM3 form complexes, where ERp46 enhances EDEM3 activity [121,122]. ERdj5, the sole human PDI with a DnaJ domain, reduces misfolded proteins in the ER, allowing for disulphide reversal in ERAD substrates [123,124]. ERdj5 also interacts with EDEM3 and BiP, presumably to facilitate mannose trimming and unfolding of various ERAD substrates before retrotranslocation [123,125–127]. To facilitate ERAD, ERdj5 must be reduced after acting on an ERAD substrate; this requires partnering with an electron donor like Ero1α [128]. There is growing evidence that multiple PDI family members participate in ERAD, specifically by partnering with mannose-trimming enzymes and reducing proteins prior to retrotranslocation into the cytosol. Given the diversity within this family, other PDI isoforms probably function in ERAD in additional capacities.

### (c) Functions of Mia40 in proteostasis

Operating in a similar fashion to the PDIs, Mia40 influences proteostasis in the mitochondrial intermembrane space. Upon protein import, Mia40 recognizes exposed hydrophobic patches in clients before facilitating structural rearrangements that lead to disulphide transfer and folding of the substrate [129–133]. Such chaperone activity can occur independently of cysteine oxidation, as some clients that lack cysteine (either naturally or by mutagenesis) are dependent on Mia40 for folding [134,135]. Moreover, several Mia40 clients, like the small Tim proteins, themselves function as chaperones [46]. These proteins form multimeric complexes with one another (Tim9/Tim10, Tim9/Tim10/Tim12 and Tim8/Tim13) after oxidation by Mia40, contributing to the folding and incorporation of proteins within the outer and inner mitochondrial membranes [136]. If proteins are mis-oxidized by Mia40, they are exported into the cytosol for degradation by the proteasome, establishing a link between Mia40 activity and proteostasis both within mitochondria and in the cytosol [137,138]. While the mechanistic details of this partnership between Mia40 activity and the proteasome need further elucidation, Mia40 both directly and indirectly influences multiple protein quality control steps in the intermembrane space.

### (d) Functions of disulphide bond-forming proteins in proteostasis

Dsb proteins contribute to proteostasis in the oxidizing confines of the bacterial periplasm by promoting the folding of proteins involved in envelope assembly and function [139]. Along these lines, both DsbA and DsbC exhibit holdase and foldase activity *in vitro* [140,141]. Moreover, loss of DsbA, in particular, impairs folding of specific proteins that function at the cell periphery, leading to induction of genes involved in protein quality control, altered secretion of proteins and decreased survival under various stress conditions [142]. Similarly, other protein folding defects and alterations in both membrane permeability and growth have been observed in *E. coli* deficient in both DsbA and DsbC [143]. Taken together, these findings highlight a critical role for the Dsb system in periplasmic proteostasis. However, little is currently known regarding whether Dsb proteins function in collaboration with other proteostasis factors that reside in the periplasm.

## 6. Outlook—defining the reach of disulphide maintenance enzymes in proteostasis

Here, I have highlighted several ways wherein specific classes of disulphide maintenance enzymes support proteostasis. These enzymes can maintain client/substrate proteins in their native ‘redox’ state (or assist them in reaching it), regulate the activity of redox-sensitive chaperones, carry out redox-independent roles as holdases and partner with other components of the proteostasis network (e.g. various chaperones and the proteasome), thereby supporting proper protein folding and damaged protein clearance. Given this diverse range of functions and the unique chemistries these enzymes employ, disulphide maintenance enzymes occupy a special, and specialized, place in the proteostasis network. It is probable that proteins with similar functions and belonging to the same evolutionary superfamilies contribute in additional ways to proteostasis. To illustrate this point, the thioredoxin superfamily is vast, with the activities of many proteins in this group remaining uncharacterized, even in well-studied model organisms. For instance, some of these proteins may recognize and remove additional reversible cysteine modifications (like those caused by hydrogen sulphide and coenzyme A, which have garnered increased attention in recent years) [144,145]. The impact of such modifications on proteostasis and the systems that cells use to reverse these less-characterized cysteine modifications remain largely undefined. Determining whether known or new disulphide maintenance enzymes aid in reversing the impact of these protein modifications will be a key area for future study. More work is also needed in identifying the full range of connections between specific disulphide maintenance systems, irrespective of the oxidative modifications they act upon, and the proteostasis network as a whole.

Separately, a growing body of evidence suggests that disulphide maintenance systems collaborate across subcellular compartments. For instance, redox cross-talk occurs between the cytoplasmic thioredoxin and DsbC in Gram-negative bacteria [146,147], between the cytosolic thioredoxin system and protein quality control machineries in the ER [148], and between cytosolic thioredoxin and Mia40 systems [149]. However, many of the mechanistic details of this intercompartmental redox collaboration need elucidation, especially in eukaryotes. Understanding redox cross-talk throughout the cell will yield a more fully integrated picture of how disulphide maintenance systems contribute to proteostasis on a holistic level.

## Data Availability

This article has no additional data.

## Theme

One contribution of 20 to a Theo Murphy meeting issue ‘ProteostaSys: a systems view of proteostasis’.
